# Air pollution and adverse birth outcomes: a narrative review of epidemiological and mechanistic findings

**DOI:** 10.1007/s40201-026-00990-4

**Published:** 2026-06-26

**Authors:** Omar Hahad, Marin Kuntic, Sadeer Al-Kindi, Jos Lelieveld, Yafang Cheng, Volker H. Schmitt, Lukas Hobohm, Karsten Keller, Jasmin Ghaemi Kerahrodi, Sasan Faridi, Nuschin Morakkabati-Spitz, Achim Fieß, Andreas Daiber, Thomas Münzel, Michelle Bous, Sybelle Goedicke-Fritz, Michael Zemlin, Nasenien Nourkami-Tutdibi, Erol Tutdibi

**Affiliations:** 1 Department of Cardiology, University Medical Center of the Johannes Gutenberg University Mainz, Langenbeckstraße 1, Mainz, 55131 Germany; 2 German Center for Cardiovascular Research (DZHK), Partner Site Rhine-Main, Mainz, Germany; 3 Center for Health and Nature, Houston, TX USA; 4 Atmospheric Chemistry Department, Max Planck Institute for Chemistry, Mainz, Germany; 5 Minerva Research Group, Max Planck Institute for Chemistry, Mainz, Germany; 6 Center for Thrombosis and Hemostasis (CTH), University Medical Center of the Johannes Gutenberg University Mainz, Mainz, Germany; 7 Department of Sports Medicine, Medical Clinic VII, University Hospital Heidelberg, Heidelberg, Germany; 8 Department of Psychosomatic Medicine and Psychotherapy, University Medical Center of the Johannes Gutenberg University Mainz, Mainz, Germany; 9 Center for Air Pollution Research (CAPR), Institute for Environmental Research (IER), Tehran University of Medical Sciences, Tehran, Iran; 10 Department of Environmental Health Engineering, School of Public Health, Tehran University of Medical Sciences, Tehran, Iran; 11 Department of Radiology, Gemeinschaftskrankenhaus Bonn, Bonn, Germany; 12 Department of Ophthalmology, University Medical Center of the Johannes Gutenberg University Mainz, Mainz, Germany; 13 Hospital for General Pediatrics and Neonatology, Saarland University Medical Center, Homburg/Saar, Germany

## Abstract

Air pollution remains a significant global health challenge and is increasingly recognized as a critical exposomic risk factor for adverse birth outcomes. Although numerous epidemiological studies have linked prenatal air pollution exposure to low birth weight, preterm birth, and stillbirth, important uncertainties remain regarding the underlying biological mechanisms, critical exposure windows, and the interplay between different pollutants and susceptibility factors. This narrative review synthesizes epidemiological findings and mechanistic evidence identified through literature searches in PubMed, Scopus, and Web of Science to provide a comprehensive overview of how maternal exposure to air pollutants affects fetal development and pregnancy outcomes. The reviewed epidemiological evidence largely supports an association between maternal air pollution exposure and adverse birth outcomes. For example, a 10 µg/m^3^ increase in fine particulate matter (PM_2.5_) exposure during the second trimester has been associated with an 11.8 g reduction in birth weight and a 23.1% increase in the risk of preterm birth. Oxidative stress, inflammation, endocrine disruption, vascular dysfunction, and epigenetic modifications are considered key biological pathways through which air pollution may impair placental function, alter fetal growth trajectories, and increase the likelihood of pregnancy complications. The placenta serves as a critical interface between maternal and fetal health and is particularly vulnerable to environmental insults, with air pollution exposure linked to changes in placental morphology, perfusion, and metabolic function. However, challenges persist in disentangling the effects of individual pollutants, establishing causality, identifying critical windows of susceptibility, and determining the extent to which sociodemographic, lifestyle, and genetic factors modify these associations. Current research gaps underscore the need for studies integrating high-resolution exposure assessment, multi-pollutant modeling, and mechanistic investigations to better clarify the impact of air pollution on pregnancy outcomes.

**Graphical Abstract:**

Air pollution and adverse birth outcomes

**Figure:**
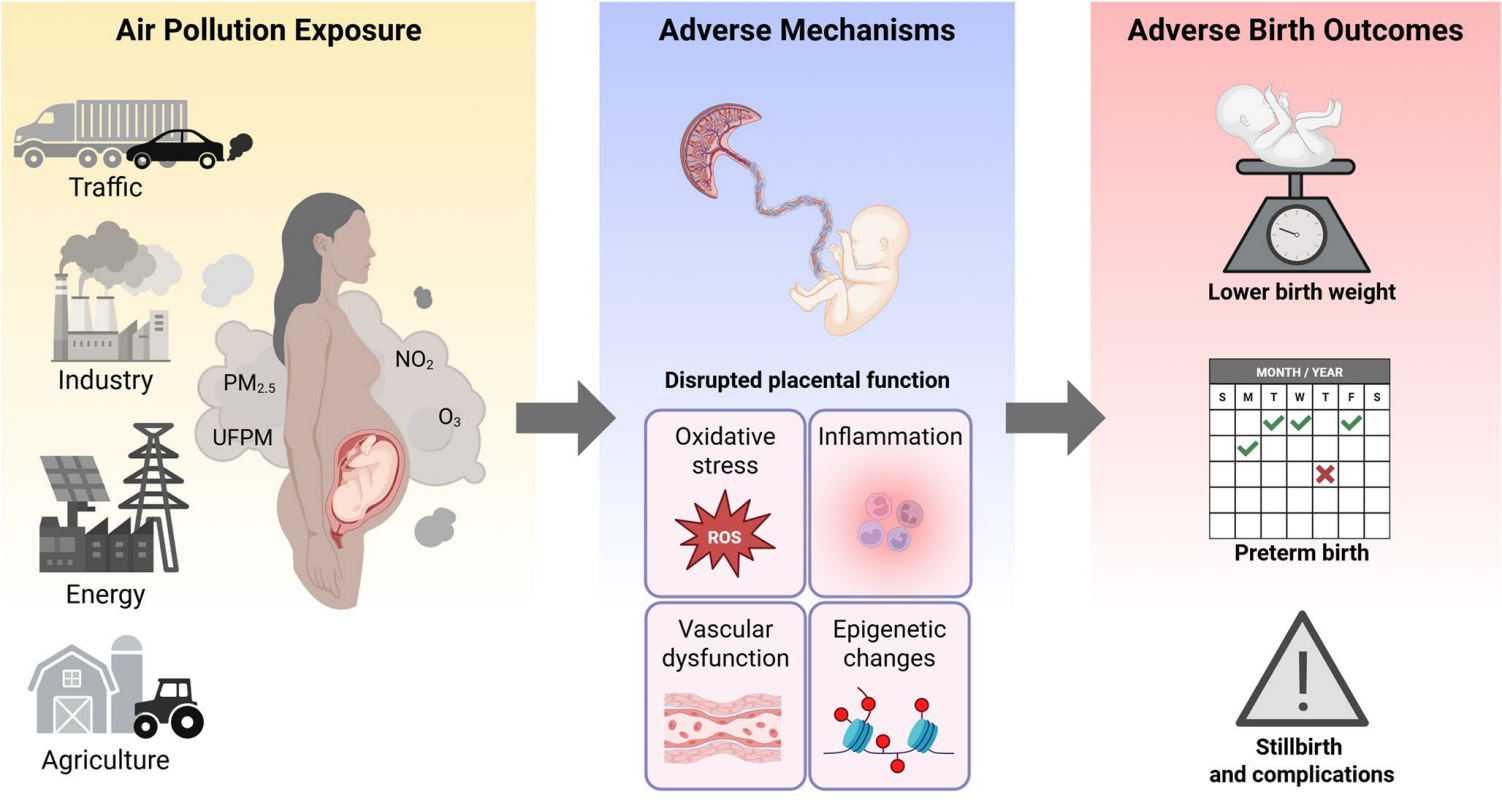

## The exposome and its role in health and disease

**Fig. 1:**
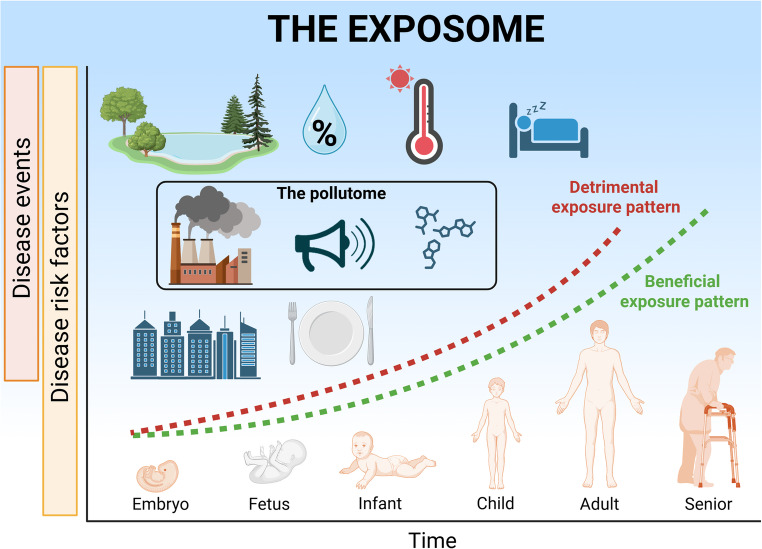
The exposome concept. The exposome encompasses the totality of environmental exposures across the life course, including chemical, physical, biological, social, and lifestyle-related factors. The illustrated trajectories represent different exposure profiles associated with lower or higher health risks over time. Disease risk factors refer to determinants that increase susceptibility to disease, whereas disease events represent the manifestation of adverse health outcomes. Their arrangement reflects the concept that cumulative environmental exposures may contribute to disease risk factors, which can subsequently lead to disease events later in life. Modified from [6] with permission. Created with BioRender.com

The concept of the exposome, introduced in 2005 by Wild [1], encompasses the lifelong physiological and pathophysiological changes induced by environmental exposures [2–4]. These exposures include a wide range of factors such as chemical pollutants, physical stressors like noise and ultraviolet radiation, as well as socioeconomic and mental health determinants like social environment, infectious agents, and psychosocial stress [5] (Fig. 1). Unlike lifestyle factors, which individuals can actively influence, the general external environment is beyond individual control. The exposome approach shifts away from analyzing single exposures leading to single health outcomes to recognizing that multiple exposures often coincide and contribute to various health outcomes. Importantly, the exposome itself should be understood as the totality of environmental exposures throughout life rather than as a construct that is inherently beneficial or harmful. Instead, specific exposures or exposure patterns may exert protective or adverse effects depending on their nature, timing, intensity, and interaction with individual susceptibility factors.

Landrigan et al. coined the term pollutome to describe the subset of the exposome that comprises all forms of pollution posing risks to human health, including chemical pollutants as well as non-chemical stressors such as light and noise pollution [7]. Whereas the exposome encompasses all environmental exposures across the life course, the pollutome specifically focuses on pollution-related exposures. The pollutome may vary across geographical locations and life stages, reflecting temporal and spatial differences in pollution exposure and their potential health effects [7]. However, our understanding of pollutomes, particularly their effects on cardiometabolic health, remains limited due to a lack of comprehensive multi-pollutant studies. Consequently, the current estimate of premature deaths and reduced healthy life expectancy attributable to chemical pollution, including air pollution, may be conservative. Many effects of pollutomes, especially those arising from emerging pollutants, are poorly understood and not adequately represented in global health studies like the Global Burden of Disease Study, underscoring the need for further research.

In recent years, the role of environmental factors, particularly air pollution, in adverse birth outcomes has become increasingly evident [6]. Vulnerable populations such as children, the elderly, and pregnant women are especially susceptible to the adverse health effects of air pollution [8–10]. Adverse birth outcomes, including low birth weight, preterm birth, and small-for-gestational-age, have been associated with neonatal complications, childhood health problems, and long-term consequences in adulthood [11]. This narrative review aims to provide a comprehensive synthesis of epidemiological evidence and mechanistic insights on the association between maternal air pollution exposure and adverse birth outcomes. It extends previous work by integrating epidemiological findings with emerging mechanistic evidence on oxidative stress, inflammation, endocrine disruption, vascular dysfunction, and epigenetic regulation, thereby providing a broader exposome-based perspective on how air pollution may affect fetal development and pregnancy outcomes. For this purpose, literature searches were conducted in PubMed, Scopus, and Web of Science using combinations of the terms “air pollution”, “particulate matter”, “PM_2.5_”, “PM_10_”, “nitrogen dioxide”, “NO_2_”, “ozone”, “pregnancy”, “placenta”, “birth outcomes”, “preterm birth”, “low birth weight”, “small-for-gestational-age”, and “stillbirth”. Additional relevant publications were identified through manual screening of reference lists from eligible articles and previous reviews.

## Air pollution as a major environmental health risk

Air pollution, as defined by the World Health Organization (WHO) as the contamination of the indoor or outdoor environment by any chemical, physical, or biological agent that alters the natural characteristics of the atmosphere, presents a significant challenge to global public health [12]. It manifests as a heterogeneous mixture of particles and gases, stemming from various human-made and natural sources. Anthropogenic activities, particularly industrial processes and the combustion of fossil fuels, play an important role in emitting pollutants into the atmosphere. Moreover, natural phenomena such as wildfires, volcanic eruptions, and dust storms also contribute particles and gases to the composition of air pollution. Although research has primarily focused on anthropogenic air pollution, emerging evidence suggests that naturally occurring particulate matter (PM) from desert dust and dust storms may also adversely affect pregnancy outcomes. A prospective cohort study from Guadeloupe reported an increased risk of preterm birth associated with maternal exposure to Saharan dust episodes [13]. Similarly, a large population-based study from South Korea linked exposure to Yellow Dust-related air pollution during pregnancy to lower birth weight, shorter gestational age, and an increased risk of low birth weight [14]. The combustion of fossil fuels, particularly in the transportation sector, emits nitrogen oxides (NO_x_=·NO_2_+·NO) and carbon monoxide (CO). Sulfur dioxide (SO_2_) primarily originates from the combustion of sulfur-containing fossil fuels, e.g., for heating and power generation, while tropospheric ozone (O_3_) forms through photochemical reactions involving NO_x_ and volatile organic compounds (VOCs). PM pollution encompasses a wide array of substances originating from various primary sources such as traffic, energy generation, industrial processes, domestic energy use, construction activities, fires, and waste incineration, as well as secondary formation through gas-to-particle conversion in the atmosphere. PM is often categorized based on the size of particles, such as inhalable PM (PM_10_), fine PM (PM_2.5_), and ultrafine PM (PM_0.1_), where the lowercase numbers indicate the upper diameter limit of particles in micrometers [15–18].

**Fig. 2:**
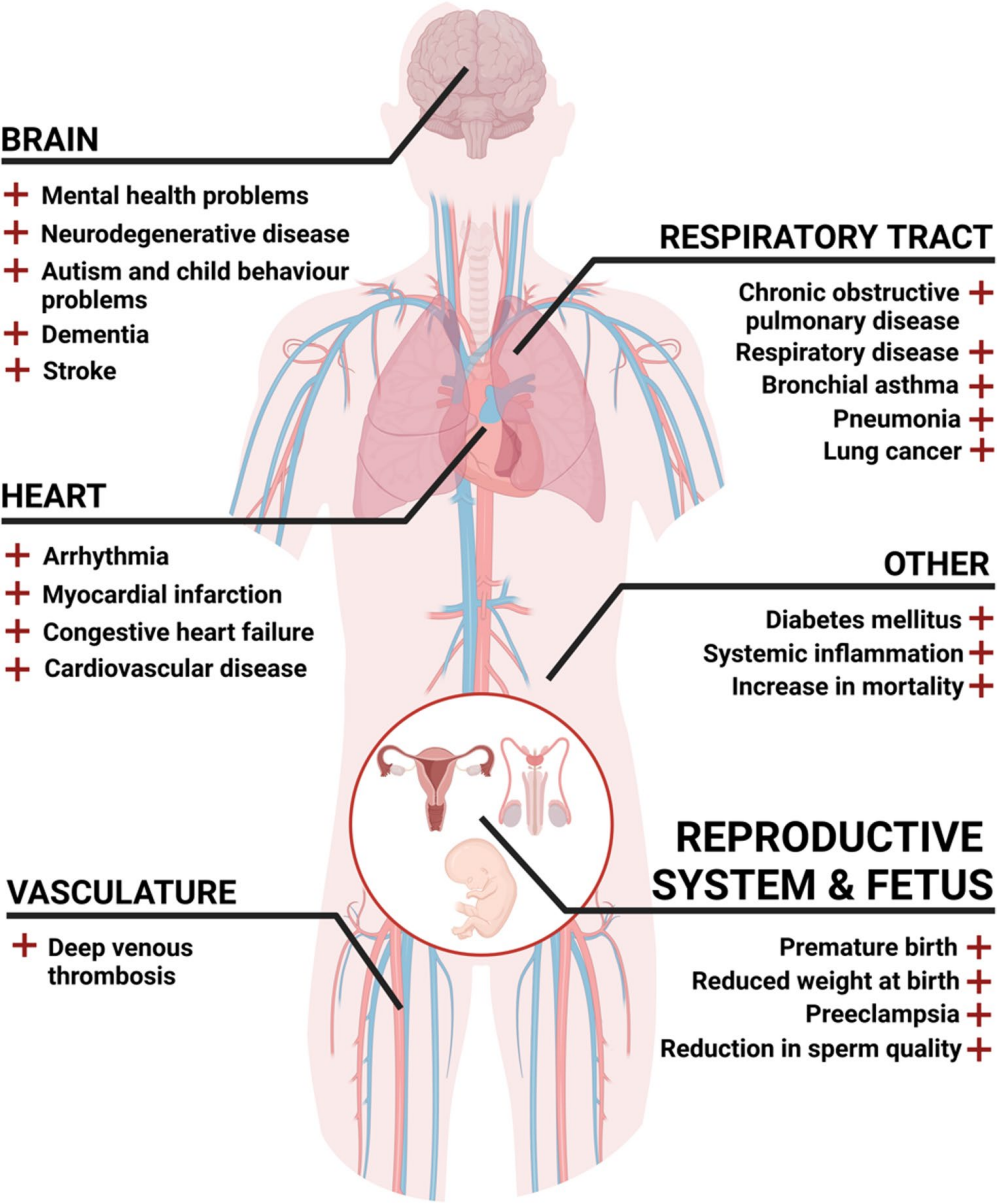
Impact of air pollution on different organ systems, including the reproductive system and the fetus. Modified from [24] with permission. Created with BioRender.com

The Lancet Commission on Pollution and Health emphasized that deteriorating air quality is the primary environmental factor driving the global disease burden and premature mortality [7] (see Fig. 2 for a comprehensive list of health conditions associated with air pollution). According to the European Environment Agency (EEA), air pollution is the leading environmental health risk factor in Europe [19]. Ambient air pollution reduces global life expectancy by 2.9 years, surpassing the impact of tobacco smoking (2.2 years) [20]. Recent assessments indicate that in 2020 alone, nine million premature deaths worldwide were associated with air pollution, primarily from PM_2.5_ [21, 22]. The EEA notes that less than 1% of the urban population in the European Union (EU) is exposed to PM_2.5_ concentrations exceeding EU standards (25 µg/m^3^) [19]. However, 97% of them are exposed to levels surpassing the new WHO guidelines established in 2021. These guidelines state that annual PM_2.5_ concentrations should not exceed 5 µg/m^3^, with 24-hour averages staying below 15 µg/m^3^ for no more than 3 to 4 days per year [23]. Recent research highlights the need to phase out fossil fuels. Fossil fuel-related ambient air pollution, including PM_2.5_ and O_3_, is estimated to cause 5.13 million excess deaths globally each year. Transitioning to clean, renewable energy sources could prevent these deaths [22].

## Adverse birth outcomes and their impact

Adverse birth outcomes encompass a range of multifactorial health issues that significantly impact pregnancy and newborn infants [25]. These outcomes typically include preterm birth, low birth weight, stillbirth, macrosomia, congenital anomalies, and infant/neonatal death [26]. Preterm birth, defined as delivery before 37 completed weeks of gestation, stands as the primary cause of neonatal mortality [27], while low birth weight identifies infants weighing less than 2500 g (approximately 5.5 pounds) at birth [28]. Conversely, stillbirth refers to fetal demise that occurs in the womb after 28 weeks of gestation [29]. Over the past four decades, there has been a significant global reduction in adverse birth outcomes. Nevertheless, the burden remains substantial, with approximately 15 million premature births and nearly 3 million stillbirths occurring annually worldwide, with 98% of these stillbirths occurring in developing countries [30–32]. Hence, the prevalence of adverse birth outcomes varies geographically and is influenced by factors such as maternal age, socioeconomic status, access to and quality of healthcare services, and underlying medical conditions [33]. Moreover, more than 45% of deaths among children under the age of five occur within the first 28 days of life [34].

**Fig. 3:**
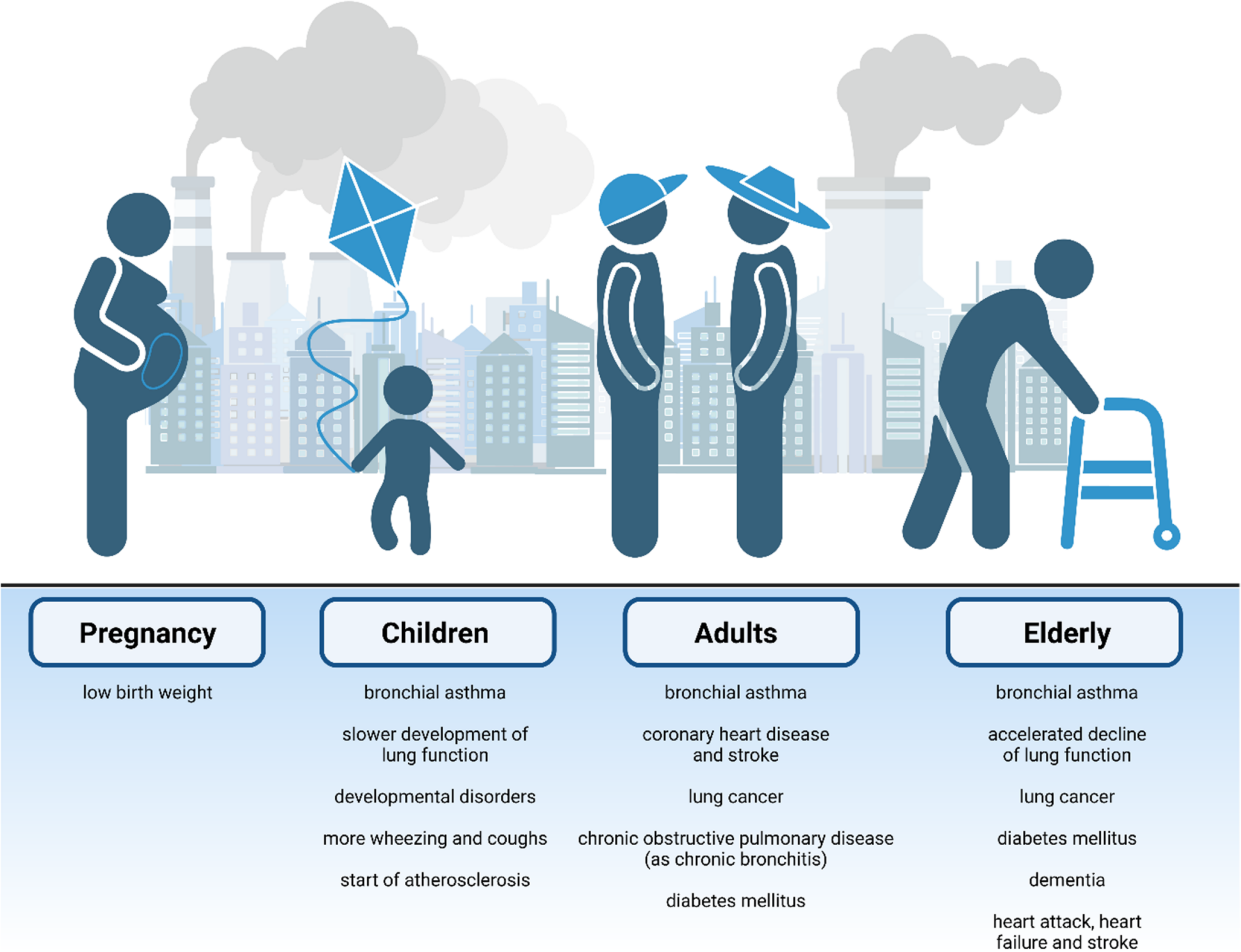
Lifelong health effects of maternal air pollution exposure. Modified from [40] with permission. Created with BioRender.com

Infants born with one or more adverse birth outcomes face heightened risks of mortality and a spectrum of health and developmental challenges [32]. These challenges often manifest as respiratory, immunological, neurological, hormonal, and behavioral complications [32, 35]. Notably, preterm birth and low birth weight are important determinants of child survival, as well as factors contributing to disabilities, stunting, and long-term adverse health consequences [36]. Infants born with low birth weight are susceptible to various complications, including heart conditions, anemia, chronic lung disorders, growth retardation, impaired cognitive development, and an increased risk of later-life metabolic disorders such as insulin resistance, metabolic syndrome, and type 2 diabetes mellitus [37]. Similarly, preterm birth subjects infants to physical and neurological difficulties that may persist as lifelong disabilities [38], with preterm complications alone accounting for approximately 27% of neonatal deaths annually, totaling about four million worldwide [39]. Therefore, maternal air pollution exposure may have lifelong effects on newborn health (Fig. 3).

## The pathophysiology of air pollution-induced diseases

Oxidative stress and inflammation are key mechanisms underlying the adverse health effects of air pollution exposure. Recent findings from both human and animal studies indicate that exposure to various air pollutants can elevate systemic oxidative stress and inflammation, thereby influencing disease risk and progression [17, 41]. However, fully understanding these pathophysiological processes remains challenging, particularly given the complex interactions with other risk and lifestyle factors such as physical activity [16, 42, 43]. While research has historically focused on O_3_ [44, 45], recent attention has shifted to PM_2.5_. However, gaseous constituents such as ·NO_2_ and SO_2_ continue to play a significant role in air pollution-induced pathophysiology [46–50]. Notably, some studies found improvements in birth outcomes, such as an increase in newborn weight and length, following maternal exposure to ·NO_2_ [51]. However, the underlying mechanism remains unclear and may involve potential confounders.

Recent clinical studies highlight both short- and long-term impacts of air pollution components on markers of oxidative stress and inflammation. PM_2.5_ exposure has been linked to oxidative damage to DNA, as indicated by elevated levels of 8-oxo-2’-deoxyguanosine (8-oxodG) [52]. Similar findings have been reported in other cohorts including Prague bus drivers and garagemen, as well as in patients with chronic obstructive pulmonary disease [53, 54]. Additionally, a positive correlation was found between atmospheric polycyclic aromatic hydrocarbons (PAHs) and malondialdehyde levels, an oxidative stress marker, in a cohort of patients with obstructive pulmonary disease and healthy controls [55]. Inflammatory markers such as interleukin-6, C-reactive protein, and white blood cell count were frequently associated with PM exposure [56–59]. Other oxidative stress and inflammation markers have also been associated with PM_2.5_ exposure [60]. A significant source and target of oxidative stress is air pollution-induced mitochondrial damage and dysfunction, which has been observed in human studies [61–64].

**Fig. 4:**
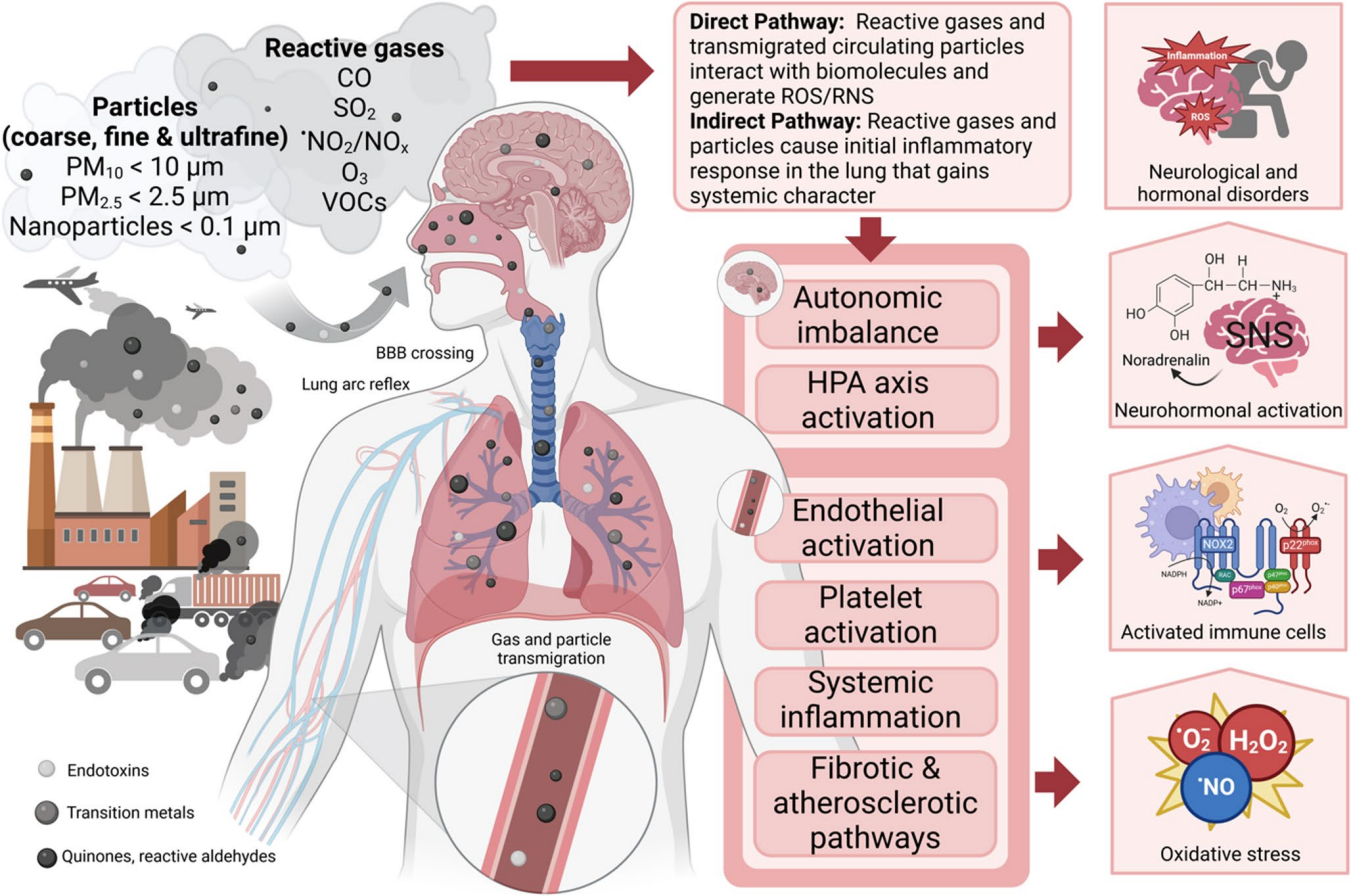
Mechanisms of air pollution-induced oxidative stress and inflammation. Pollutant gases and PM enter the body through the pulmonary system, translocating into circulation or directly reaching the nervous system via the olfactory nerves. Once inside, air pollution triggers oxidative stress and inflammation in distant organs, contributing to neurological and hormonal disorders. Modified from [17] with permission. Created with BioRender.com

Most of the current understanding of the molecular mechanisms of air pollution-induced oxidative stress and inflammation comes from animal studies, as human and cell culture experiments are limited to controlled exposures. Two primary pathways have been identified by which air pollution, particularly PM, can induce oxidative stress and inflammation in remote organs. The first is direct: ultrafine PM can cross the air-blood barrier, enter the circulation, and reach distant tissues [65, 66]. Studies have shown that resident liver macrophages can internalize PM, leading to liver inflammation and fibrosis [67]. This pathway is particularly detrimental to the cardiovascular system, as PM can directly damage the endothelial layer of blood vessels, promoting atherosclerosis [68, 69]. Ultrafine PM can also enter the central nervous system through the olfactory bulb [70], where it may induce the release of pro-inflammatory mediators, compromise the blood-brain barrier (BBB), and trigger neurohormonal stress responses [71–75]. The second pathway is indirect and involves an inflammatory response initiated the lungs [76, 77]. The activated immune system releases pro-inflammatory cytokines that enter the circulation, leading to systemic inflammation. This, in turn, triggers oxidative stress in remote organs, primarily driven by activated immune cells [78, 79]. The indirect pathway is studied more extensively than the direct pathway, as circulating cytokines and systemic inflammation markers are easier to assess than direct PM-induced tissue damage. The mechanisms of air pollution-induced oxidative stress and inflammation are displayed in Fig. 4.

### The pathophysiology of air pollution-induced adverse birth outcomes

**Fig. 5:**
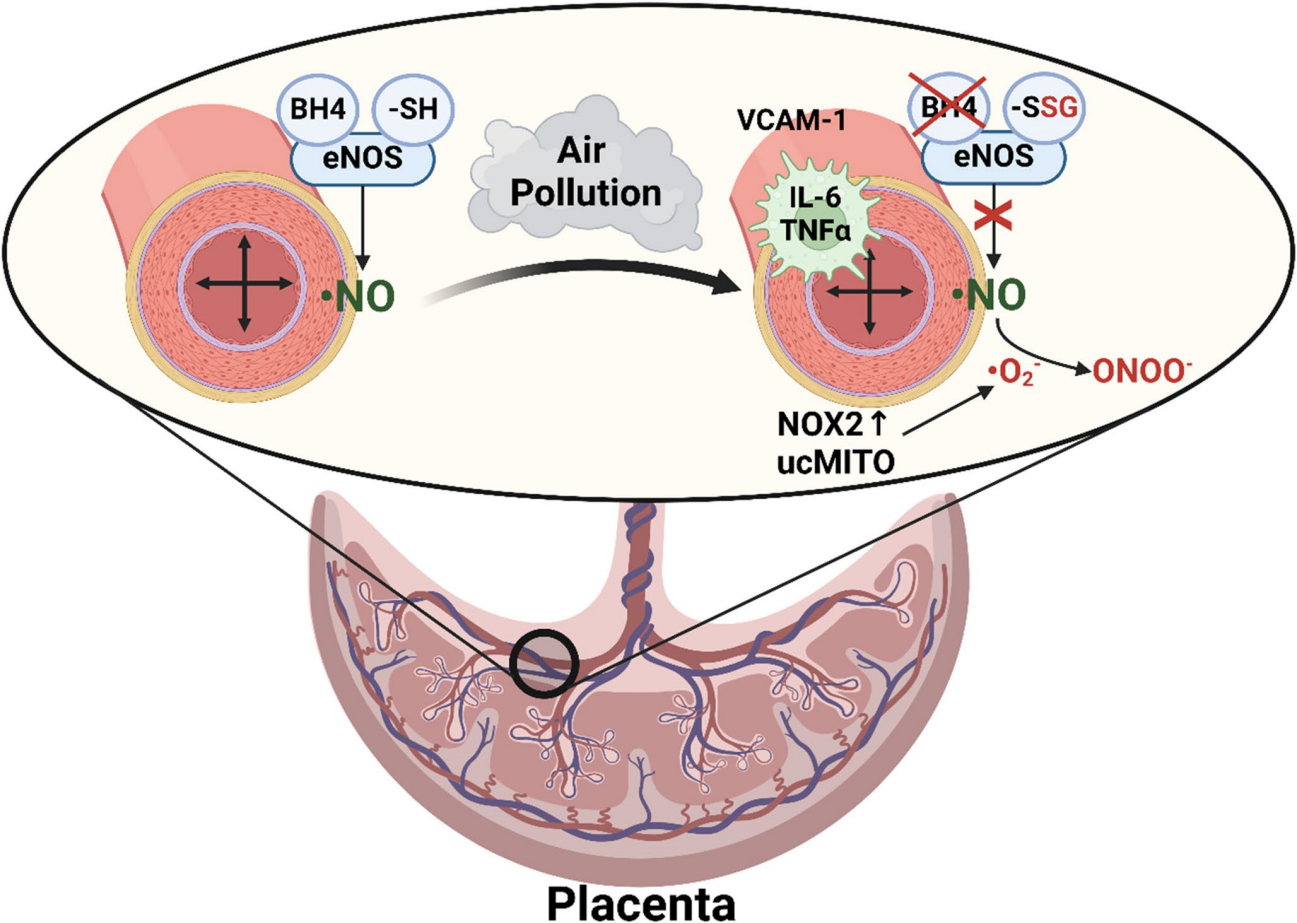
Major pathophysiological alterations in the placental system induced by air pollution exposure. Endothelial nitric oxide (·NO) synthase (eNOS), tetrahydrobiopterin (BH4), vascular cell adhesion molecule 1 (VCAM-1), NADPH oxidase subunit ·NOX2, uncoupled mitochondria (ucMITO), Interleukin 6 (IL-6), tumor necrosis factor alpha (TNFα). Created with BioRender.com

**Fig. 6:**
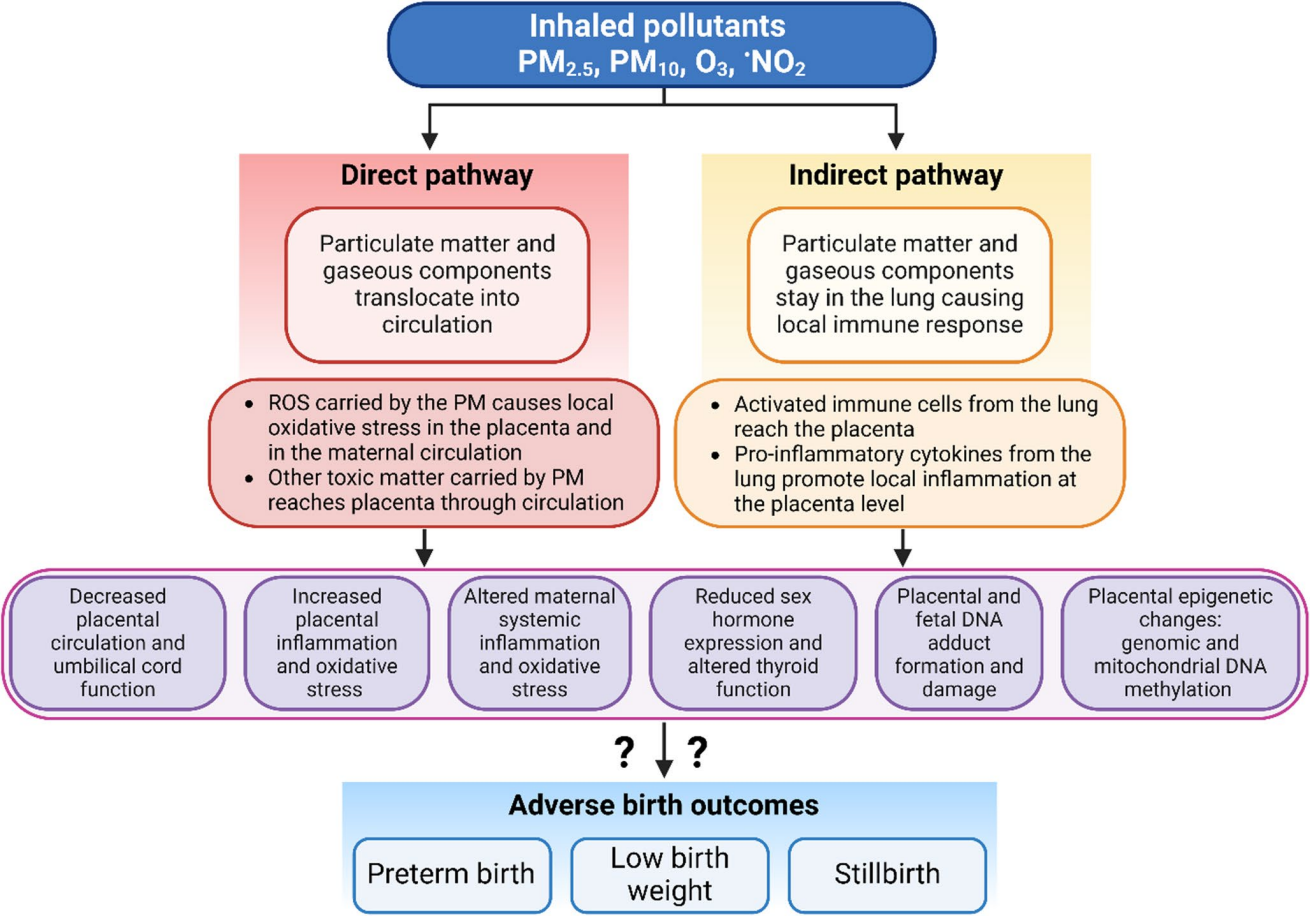
Mechanisms by which air pollution contributes to adverse birth outcomes. Modified from [80] with permission. Created with BioRender.com

Like other diseases affecting remote organ systems, such as the cardiovascular or the nervous systems, the reproductive system is also vulnerable to air pollution-induced oxidative stress and inflammation. However, the impact of air pollution on adverse birth outcomes is multifaceted, involving a complex interplay of various factors during gestation, which may also have lasting effects across generations [80]. The most studied factors are related to the placenta, the organ regulating and supporting fetal growth. The placenta is susceptible to nitrosative/oxidative stress, inflammation, endocrine disruption, epigenetic alterations, and vascular dysregulation within the maternal-fetal unit, all of which can lead to impaired fetal growth and adverse birth outcomes [81]. The placenta can also sustain significant damage from preeclampsia (severe hypertension during pregnancy), which accounts for approximately one-third of all very preterm births [82]. Preeclampsia is highly related to oxidative stress, endothelial dysfunction, and impaired perfusion of the placenta [83]. In concordance, preeclampsia benefits from therapy with pentaerythritol tetranitrate [84], an antioxidant vasodilator and epigenetic modulator, or L-citrulline [85], a precursor of the endothelial nitric oxide (·NO) synthase (eNOS) substrate L-arginine. These compounds exert antioxidant, anti-inflammatory, and vasculoprotective effects. Endothelial dysfunction that is associated with preeclampsia is driven by eNOS uncoupling [82]. eNOS uncoupling occurs when its cofactor tetrahydrobiopterin (BH_4_) or its substrate L‑arginine are depleted, or when the enzyme is altered by redox‑dependent post‑translational modifications, so that instead of transferring electrons to L‑arginine to generate ·NO, it transfers them directly to molecular oxygen and generates superoxide, driving oxidative stress. Oxidative stress oxidizes or depletes BH₄ and can lower effective L‑arginine availability or increase endogenous NOS inhibitors such as asymmetric dimethylarginine (ADMA), all of which destabilize the eNOS dimer and favor the uncoupled state. In parallel, oxidative modifications like S‑glutathionylation, as well as adverse phosphorylation patterns and disrupted protein–protein interactions, further driving eNOS uncoupling [86]. The major pathophysiological alterations in the placental system induced by air pollution exposure are displayed in Figs. 5 and 6.

#### Oxidative stress

Oxidative stress is a key mechanism underlying air pollution-induced adverse birth outcomes [87]. Oxidative stress is primarily mediated by reactive oxygen and nitrogen species (RONS), which can originate directly from air pollution – such as O_3_, ·NO_2_, and free radicals present in PM – or from enzymatic processes disrupted by air pollution components [61, 88]. Placental oxidative stress has been linked to PM_2.5_ exposure, as evidenced by increased levels of 3-nitrotyrosine (3-NT) [89]. 3-NT is a protein modification resulting from peroxynitrite (ONOO^-^), a reactive molecule formed by the reaction between nitric oxide (·NO) and the superoxide radical (·O_2_^-^) [90]. ·NO plays a crucial role in vascular signaling, particularly in the development of the placenta and umbilical cord [82]. Women with preeclampsia may be even more susceptible to oxidative stress. A study from Mexico demonstrated that oxidative stress markers (such as protein carbonyls and malondialdehyde) were significantly elevated in the placentas of women with preeclampsia and in their newborns who were exposed to higher ambient air pollution levels (PM_2.5_, PM_10_, and O_3_) during the first and second trimester of pregnancy [91]. In agreement, a study monitoring personalized exposure in 215 pregnant women showed that PM_2.5_ was associated with increased blood pressure and elevated plasma markers of endothelial dysfunction (endothelin-1, E-selectin, and intercellular adhesion molecule 1) [92], further supporting the role of oxidative stress. During pregnancy, maternal energy expenditure increases to meet the metabolic demands of the embryo and fetus, leading to elevated mitochondrial activity and an increased susceptibility to oxidative stress [93]. A decline in mitochondrial DNA copy number has been associated with air pollution exposure, suggesting a negative impact on cellular energy production [94–97]. Additionally, oxidative damage to mitochondrial DNA in umbilical cord blood and placental tissue, indicated by elevated 8-oxodG levels, has been positively correlated with PM_2.5_ and PM_10_ exposure [64, 98]. A study in pregnant women living in highly industrialized, polluted areas showed increased mitochondrial depolarization along with systemic oxidative stress markers, such as malondialdehyde [99]. Interestingly, superoxide dismutase 2 (SOD2) expression was upregulated in highly exposed women. However, the expression of nuclear factor erythroid 2-related factor 2 (Nrf2) and glutathione was decreased, indicating an activation of mitochondrial antioxidant defenses while cytosolic antioxidant mechanisms remained impaired.

#### Inflammation

Inflammation and oxidative stress are closely interconnected, as activated immune cells release large amounts of ROS to combat pathogens [100]. During blastocyst implantation and placentation through trophoblast invasion, the maternal immune system plays a crucial role by providing protection and facilitating molecular signaling [101–104]. Exposure to air pollution components such as PM_2.5_, PM_10_, ·NO_2_, and O_3_ has been linked to increased levels of inflammation markers, including C-reactive protein (CRP), interleukin-1β, interleukin-6 (IL-6), and tumor necrosis factor alpha (TNF-α) in both maternal and umbilical cord blood [105–107]. Elevated maternal levels of CRP, TNF-α and IL-6 are also associated with adverse birth outcomes, such as preterm birth and fetal growth restriction [108, 109]. However, the direct relationship between air pollution and these outcomes remains unclear [110]. One study demonstrated that IL-6 expression in the placenta was specifically associated with PM_2.5_ exposure during the first trimester in women with clinically recognized early pregnancy loss but not in those with normal pregnancies. However, both groups demonstrated a dose-dependent increase in inflammatory markers linked to PM_2.5_ exposure [111]. Additionally, PM_10_ has been associated with higher levels of soluble vascular cell adhesion molecule 1 (sVCAM-1) and plasminogen activator inhibitor 1 (PAI-1) in the circulation of pregnant women, suggesting increased systemic inflammation and potential endothelial dysfunction [112]. In a preeclampsia rat model, PM_2.5_ exposure was linked to elevated mRNA and protein levels of TNF- α, monocyte chemoattractant protein 1 (MCP1), macrophage inflammatory protein 1-alpha (MIP-1-alpha) and C-C chemokine receptor type 1 (CCR1) [113]. Similarly, a mouse model demonstrated that nasal instillation of PM_2.5_ triggered placental inflammation and reduced placental vascular cell count [114]. Although it is well-established that air pollution components influence inflammatory processes in both the mother and the fetus, further research is required to better understand their associations with adverse birth outcomes.

#### Changes in epigenetic regulation

Epigenetic modifications and adaptation play a crucial role in the formation and maintenance of the placenta as well as fetal growth by regulating the expression of genes essential for developing tissues [115]. Global DNA methylation has been observed to decrease in umbilical cord blood and placenta tissue in women exposed to higher levels of PM_2.5_, as well as elevated levels of O_3_ and ·NO_2_ [116–120]. In addition to global changes, locus-specific alterations in DNA methylation have also been reported. For instance, HSD11B2 (involved in glucocorticoid metabolism) and H19 (important for fetal growth), were found to exhibit altered methylation patterns in response to increased air pollution exposure [51, 117]. A study on placental DNA methylation showed that methylation of *ADORA2B*, a gene associated with hypoxia and preeclampsia [121], was positively associated with ·NO_2_ and PM_10_ exposure [122]. Interestingly, PM_2.5_ exposure was associated with altered DNA methylation in promoter regions regulating circadian clock gene transcription [123]. Mitochondrial DNA methylation at the D-loop control region was also observed in placental tissue and positively correlated with PM_2.5_ exposure [124]. This methylation pattern, previously linked to smoking during pregnancy, has been associated with lower birth weight [125].

#### Disruption in endocrine signaling

Maternal hormone balance plays a critical role during embryonic development, and any disruption can result in adverse birth outcomes [80]. Several air pollution components, such as PAHs and persistent organic pollutants (POPs) found in PM, have the potential to disrupt the endocrine system. These pollutants can interfere with steroidogenesis [126, 127] and have been associated with gestational hypertension and preeclampsia [128]. Several epidemiological studies showed that different components of air pollution can reduce levels of hormones such as estradiol, progesterone, follicle-stimulating hormone, and cortisol in pregnant women and those expecting pregnancy [129, 130]. This disruption has been corroborated in mouse and cellular models exposed to PM_2.5_ [131, 132]. Non-sex hormones, such as those produced by the thyroid gland, have also been affected by air pollution exposure during pregnancy, with impacts observed both in the mothers and the offspring [133–135]. In addition, insulin levels in umbilical cord blood were positively associated with PM_2.5_ exposure, indicating a potential influence on glucose metabolism in the offspring [136]. Air pollution has also been linked to male reproductive health issues, providing another possible mechanism contributing to infertility or adverse birth outcomes [137, 138].

## Epidemiological evidence on the association between air pollution and adverse birth outcomes

Numerous epidemiological studies have investigated the association between air pollution and adverse birth outcomes (for previous reviews, see [11, 80, 139–144]. In a recent meta-analysis including 15 epidemiological studies, exposure to PM_2.5_ and CO during the third trimester of pregnancy, as well as throughout the entire pregnancy, was associated with higher odds of stillbirth [145]. However, PM_10_, SO_2_, and ·NO_2_ exposure did not affect stillbirth. Another meta-analysis including 40 studies (case-control or cohort designs) revealed odds ratios (OR) ranging from 1.03 to 1.21 for low birth weight and from 0.97 to 1.06 for preterm birth with exposure to CO, ·NO_2_, NO_x_, O_3_, PM_2.5_, PM_10_, or SO_2_ throughout pregnancy with evidence of a low risk of bias [146].

In a national study in Canada conducted between 1999 and 2008, approximately 2.5 million births were analyzed to demonstrate that ·NO_2_ exposure was associated with increased odds of small-for-gestational-age births and reduced term birth weight [147]. In a further study of 2,766 infertile patients undergoing in vitro fertilization in Shanghai from 2016 to 2019, exposure to ·NO_2_ was associated with lower pregnancy rates, while PM_10_ was linked to reduced live birth rates [148]. Distributed lag models found that gestational weeks 22–32 were a critical window when ·NO_2_ exposure had the strongest associations with small-for-gestational-age [149]. The associations of air pollution exposure tended to be stronger in female than male newborns. Patients undergoing single embryo transfer were particularly vulnerable to SO_2_ and O_3_ exposure, resulting in lower pregnancy and live birth rates. In a study involving 163,868 women with pregnancies in Ho Chi Minh City, Vietnam, exposure to PM_2.5_ was associated with lower birth weight and increased risk of preterm birth [150]. Specifically, each 10 µg/m^3^ increase in PM_2.5_ during the second trimester was linked to an 11.771 g decrease in birth weight and a 23.1% increase in the risk of preterm birth. However, there was no significant association with the term low birth weight. In a study involving 3,988 newborns born to women in 1998 in the City of Kaunas, Lithuania, each 10 µg/m^3^ increase in ·NO_2_ concentrations during the second trimester was associated with a 25% increase in the risk of preterm birth [151]. In a nested case-control survey within a birth cohort including 2,543 women from 58,316 births in 2003 in Los Angeles County, United States, higher CO and PM_2.5_ exposures during the first trimester consistently raised preterm birth odds [152]. In a study spanning from 2005 to 2012 in the Como province in Italy, data from 3,988 newborns was analyzed to demonstrate that increased second-trimester NO_x_ exposure elevated the risk of preterm birth with an OR of 1.53 (95% confidence interval (CI) 1.25–1.87) [153]. Additionally, third-trimester PM_10_ exposure increased the risk of low birth weight (OR 1.44, 95% CI 1.03–2.02). In a large UK cohort study, researchers investigated the impact of air pollution on preterm birth and fetal growth [154]. They analyzed data from 203,562 births in Northwest England from 2004 to 2008. Using novel and traditional exposure estimation techniques, results showed a small but significant association between PM_10_ exposure and small-for-gestational-age, especially in the first and third trimesters. Similar effects were observed for ·NO_2_, PM_2.5_, and CO in later pregnancy. However, no associations were found with NO_x_ for preterm birth or birth weight reduction. Using data from 10,960 pregnant women from China revealed that exposure to PM_2.5_, PM_10_, ·NO_2_, SO_2_, and CO before and during pregnancy was associated with an increased risk of preterm birth and low birth weight [155]. In contrast, using data from the Amsterdam Born Children and their Development (ABCD) study, no increased risk of preterm birth among highly exposed women to traffic-related air pollution during pregnancy was found (*N* = 7,600 births). Children of mothers with high ·NO_2_ exposure had higher average term birth weight and lower risk of being small-for-gestational-age, with no clear dose-response relationship [156]. Analyzing 423,719 births in Florida, USA, between 2004 and 2005, trimester-specific exposure levels were estimated. The results revealed that PM_2.5_ exposure in all trimesters was associated with increased risk of term low birth weight, preterm delivery, and very preterm delivery, particularly during the second trimester. Conversely, O_3_ exposure showed inconsistent effects, with positive and protective associations observed [157]. Interestingly, by analyzing data from 423,719 births in Florida from 2004 to 2005, a study could demonstrate the pronounced effects of PM_2.5_ on preterm birth among diabetic mothers, while the effects of O_3_ were heightened among mothers with bronchial asthma [158]. A Swedish cohort study analyzing 40,245 births found that exposure to PM_2.5_ from local sources, particularly small-scale residential heating, was associated with lower birth weight and an increased risk of low birth weight (OR 1.14, 95% CI 1.04–1.26) [159].

Taken together, the available epidemiological evidence largely supports an association between maternal exposure to PM and gaseous air pollutants and an increased risk of adverse birth outcomes, particularly preterm birth, low birth weight, and small-for-gestational-age. However, the magnitude and consistency of these associations vary across studies, likely reflecting differences in exposure assessment, study populations, pollutant mixtures, and potential confounding factors.

## Summary and conclusions

Prenatal exposure to air pollution remains a critical environmental health concern with substantial implications for maternal and fetal well-being. The findings presented in this narrative review underscore the important role of air pollutants, including PM and gaseous pollutants, in shaping adverse birth outcomes such as preterm birth, low birth weight, and fetal growth restriction. While epidemiological evidence largely supports these associations, inconsistencies persist due to variations in exposure assessment, population characteristics, and potential confounding factors.

Mechanistic studies highlight oxidative stress, inflammation, endocrine disruption, vascular dysfunction, and epigenetic modifications as key pathways mediating the adverse effects of air pollution on placental function and fetal development. Although the biological plausibility of these pathways is well-established [80], further research is required to disentangle the precise molecular mechanisms and their interactions with sociodemographic, lifestyle, and genetic factors.

Emerging evidence suggests that lifestyle factors, including physical activity [16, 42, 43] and nutrition [160], may modulate the adverse effects of air pollution, but their role in the specific context of pregnancy remains elusive. A recent scoping review concluded that evidence for effect modification by physical activity, nutrition, and pre-pregnancy body-mass-index remains limited, weak, and inconsistent, highlighting the need for further prospective studies [161]. Future studies should investigate whether maternal lifestyle and behavior can modify the risks associated with air pollution, potentially offering intervention strategies to protect vulnerable populations. In addition, climate change may alter exposomic profiles through increasing temperatures, more frequent wildfires, dust storms, and changes in air pollutant formation, potentially amplifying environmental risks during pregnancy [162]. Moreover, while much of the research to date has focused on PM, the health impacts of gaseous pollutants, both individually and in potential synergy with PM, warrant further investigation [68].

However, several limitations and challenges should be acknowledged. A critical aspect to consider is the heterogeneity in study quality and methodological approaches. Exposure assessment techniques, study design variations, and residual confounding factors contribute to discrepancies in observed associations. Evaluating study biases and enhancing methodological rigor through standardized exposure assessments, multi-pollutant models, and improved statistical controls will be essential for strengthening causality.

In conclusion, prenatal air pollution exposure should be regarded as an important exposomic determinant of adverse birth outcomes with potential consequences extending from fetal development into childhood and adulthood. The evidence reviewed here highlights the need to move beyond single-pollutant approaches and to consider the cumulative and interacting effects of environmental exposures within the broader exposome and pollutome frameworks. Continued interdisciplinary research integrating epidemiological, mechanistic, and exposomic approaches will be essential to better characterize critical windows of susceptibility and identify effective preventive strategies. At the same time, the implementation of stringent air quality regulations, cleaner energy transitions, and targeted protection of vulnerable populations should remain public health priorities to reduce the global burden of adverse birth outcomes and promote maternal and child health.

## Data Availability

No new data were created or analyzed in this study. Data sharing is not applicable to this article.
